# Formation of the NLRP3 inflammasome inhibits stress granule assembly by multiple mechanisms

**DOI:** 10.1093/jb/mvae009

**Published:** 2024-01-31

**Authors:** Daisuke Yoshioka, Takanori Nakamura, Yuji Kubota, Mutsuhiro Takekawa

**Affiliations:** Division of Cell Signaling and Molecular Medicine, Institute of Medical Science, The University of Tokyo, 4-6-1 Shirokanedai, Minato-ku, Tokyo 108-8639, Japan; Department of Computational Biology and Medical Sciences, Graduate School of Frontier Sciences, The University of Tokyo, Chiba 277-8583, Japan; Division of Cell Signaling and Molecular Medicine, Institute of Medical Science, The University of Tokyo, 4-6-1 Shirokanedai, Minato-ku, Tokyo 108-8639, Japan; Division of Cell Signaling and Molecular Medicine, Institute of Medical Science, The University of Tokyo, 4-6-1 Shirokanedai, Minato-ku, Tokyo 108-8639, Japan; Division of Cell Signaling and Molecular Medicine, Institute of Medical Science, The University of Tokyo, 4-6-1 Shirokanedai, Minato-ku, Tokyo 108-8639, Japan; Department of Computational Biology and Medical Sciences, Graduate School of Frontier Sciences, The University of Tokyo, Chiba 277-8583, Japan; Medical Proteomics Laboratory, Institute of Medical Science, The University of Tokyo, 4-6-1 Shirokanedai, Minato-ku, Tokyo 108-8639, Japan

**Keywords:** DHX33, NLRP3 inflammasome, poly(I:C), potassium efflux, stress granules

## Abstract

Proper regulation of cellular response to environmental stress is crucial for maintaining biological homeostasis and is achieved by the balance between cell death processes, such as the formation of the pyroptosis-inducing NLRP3 inflammasome, and pro-survival processes, such as stress granule (SG) assembly. However, the functional interplay between these two stress-responsive organelles remains elusive. Here, we identified DHX33, a viral RNA sensor for the NLRP3 inflammasome, as a SG component, and the SG-nucleating protein G3BP as an NLRP3 inflammasome component. We also found that a decrease in intracellular potassium (K^+^) concentration, a key ‘common’ step in NLRP3 inflammasome activation, markedly inhibited SG assembly. Therefore, when macrophages are exposed to stress stimuli with the potential to induce both SGs and the NLRP3 inflammasome, such as cytoplasmic poly(I:C) stimulation, they preferentially form the NLRP3 inflammasome but avoid SG assembly by sequestering G3BP into the inflammasome and by inducing a reduction in intracellular K^+^ levels. Thus, under such conditions, DHX33 is primarily utilized as a viral RNA sensor for the inflammasome. Our data reveal the functional crosstalk between NLRP3 inflammasome-mediated pyroptosis and SG-mediated cell survival pathways and delineate a molecular mechanism that regulates cell-fate decisions and anti-viral innate immunity under stress.

## Graphical Abstract

**Graphical Abstract:**
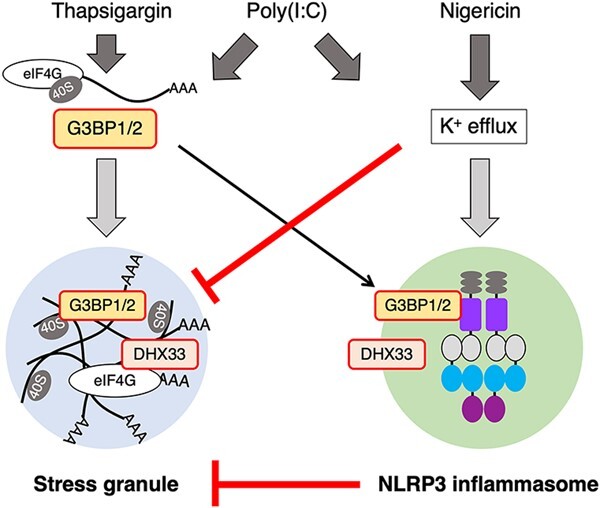

## Abbreviations

ASC: apoptosis-associated speck-like protein containing a caspase-activating recruitment domain
dsRNA: double-stranded RNA
eIF4G: eukaryotic translation initiation factor 4G
G3BP: Ras-GTPase-activating protein-binding protein
GSDMD: gasdermin D
HS: heat shock
IDRs: intrinsically disordered regions
IL-1β: interleukin-1β
ISRIB: integrated stress response inhibitor
LLPS: liquid–liquid phase separation
NLRP3: NOD-, LRR- and pyrin domain-containing protein 3
PRRs: pattern recognition receptors
RNPs: RNA-binding proteins
RPS3/6: ribosomal protein S3/S6
SA: sodium arsenite
SGs: stress granules
Tg: thapsigargin
TPA: 12-O-tetradecanoylphorbol 13-acetate

In coping with environmental stresses, mammalian cells either activate defence mechanisms to survive or initiate cell death processes, such as apoptosis and pyroptosis, depending on the type and level of stress. Such protective and deleterious signalling pathways are often interconnected, and their functional crosstalk is crucial for dictating cell-fate decisions under stress. A key cellular defence mechanism is the assembly of stress granules (SGs). SGs are membraneless organelles that rapidly form in the cytoplasm in response to certain types of stress, such as a viral infection, endoplasmic reticulum (ER) stress, arsenite and heat shock (HS) *(**1**)*. The core components of SGs are condensates of stalled 48S translation initiation complexes containing 40S ribosomal subunits (*e.g.* RPS3 and RPS6), translation initiation factors (*e.g.* eIF4G), non-translating mRNAs and many RNA-binding proteins (RBPs) such as Ras-GTPase-activating protein-binding proteins 1 and 2 (G3BP1/2) and T-cell intracellular antigen 1 (TIA-1). In addition, SGs include many other proteins involved in diverse cellular functions *(**2**,*  *3**)*. However, despite recent progress, a complete list of SG components is still lacking.

In most cases, the assembly of SGs is triggered by stress-induced phosphorylation of the α-subunit of eukaryotic translation initiation factor 2α (eIF2α) and requires the self-aggregation of specific RBPs, such as G3BP1/2 and TIA-1 *(**1**,*  *4**,*  *5**)*. In cells exposed to stress, several different stress-sensing kinases, including protein kinase R (PKR) and PKR-like ER kinase, phosphorylate eIF2α. Phosphorylated eIF2α then inhibits productive translation initiation by preventing the formation of the eIF2–GTP–tRNAi^Met^ ternary complex, which is required to load the initiator tRNAi^Met^ onto the 43S pre-initiation complex. This event leads to the assembly of a translationally stalled 48S complex and the dissociation of translating 80S ribosomes from mRNA. The released mRNA then recruits the stalled 48S complexes and SG-nucleating RBPs (*e.g.* G3BP1/2) that possess a self-interaction domain and intrinsically disordered regions (IDRs), which can promiscuously interact with various proteins and RNAs *(**6**)*. The resulting mRNA-protein complexes undergo liquid–liquid phase separation (LLPS) through RBP-mediated self-oligomerization and multivalent interactions with other SG proteins and RNAs, thereby assembling into SGs. Besides this standard eIF2α phosphorylation-dependent mechanism, some SGs are known to form without eIF2α phosphorylation *(**1**)*.

Although the precise function of SGs is not fully understood, increasing evidence suggests that SGs act as signalling hubs by incorporating multiple signalling molecules and regulate cell-fate decisions and immune responses under stress *(**1**,*  *7**)*. Indeed, previous studies, including our own, have demonstrated that SG formation inhibits several signalling pathways that promote cell death, such as p38, c-Jun N-terminal kinase (JNK), mammalian target of rapamycin (mTOR) and caspase cascades, by sequestering molecules related to these pathways (*e.g.* RACK1, Raptor, and caspases) *(**3**,*  *8**,*  *9**)*. Furthermore, SGs also play a role in the regulation of cellular responses to pathogen infection *(**10**,*  *11**)*. For instance, a recent study showed that the formation of SGs prevented the overactivation of innate immune signalling pathways that are mediated by certain pattern recognition receptors (PRRs), such as retinoic acid-inducible gene I (RIG-I) and melanoma differentiation-associated protein 5 (MDA5) *(**12**)*. Moreover, in macrophages, SGs inhibit NOD-, LRR- and pyrin domain-containing protein 3 (NLRP3) inflammasome-induced pyroptosis and cytokine production *(**13**)*. When SGs are formed, they sequester DDX3X, which is an essential component of the NLRP3 inflammasome, into the granules, thereby suppressing the assembly and activation of the inflammasome. Therefore, SGs have recently been proposed as shock absorbers that prevent excessive innate immune responses *(**12**)*.

In contrast to the adaptive roles of SGs, one of the cellular destructive responses to stress is the formation of the NLRP3 inflammasome. The NLRP3 inflammasome consists mainly of NLRP3, apoptosis-associated speck-like protein containing a caspase-activating recruitment domain (ASC) and caspase-1 and is assembled and activated when innate immune cells (*e.g.* macrophages, neutrophils and dendritic cells) are exposed to a variety of stress or infectious stimuli, including danger-associated molecular patterns (such as uric acid crystals and ATP) and pathogen-associated molecular patterns (such as viral RNA and microbial components). Although the precise mechanisms of NLRP3 inflammasome assembly remain obscure, several lines of evidence indicate that most NLRP3 agonists ultimately lead to a decrease in the intracellular potassium (K^+^) concentration, thereby inducing inflammasome activation *(**14*–*16**)*. Indeed, cellular K^+^ efflux caused by a potassium ionophore, nigericin, is sufficient to induce the assembly and activation of the NLRP3 inflammasome *(**17**)*. Therefore, a decrease in intracellular K^+^ levels is considered a common trigger for the activation of the NLRP3 inflammasome. Once the NLRP3 inflammasome is activated, pro-caspase-1 undergoes self-cleavage and activation. Activated caspase-1, in turn, cleaves and activates the pore-forming protein gasdermin D (GSDMD) and the proinflammatory cytokines, pro-interleukin 1β (IL-1β) and 18 (IL-18). The cleaved N-terminal fragment of GSDMD (N-GSDMD) forms pores in the cell membrane, thereby releasing activated cytokines (mature IL-1β and IL-18) and inducing pyroptosis to further promote the inflammatory response *(**18**)*. Therefore, activation of the NLRP3 inflammasome is crucial for the induction of pyroptotic cell death and the resulting host defence against pathogens. Although both pro-survival SGs and the pyroptosis-inducing NLRP3 inflammasome are involved in the regulation of the cellular stress response and innate immunity, their functional relationship still remains elusive.

DEAD-box (DDX) and DEAH-box (DHX) proteins form two major families of RNA helicases, named for their characteristic conserved Asp-Glu-Ala-Asp (DEAD)/Asp-Glu-Ala-His (DEAH) motifs. They play important roles not only in RNA metabolism (*e.g*. transcription, splicing, nucleocytoplasmic transport, translation and RNA decay) but also in the antiviral immune response by acting as sensors for viral nucleic acids or by regulating signal transduction downstream of various PRRs *(**19**,*  *20**)*. For instance, DDX3, DDX6, DHX9, DDX41, DDX60 and the DDX1–DDX21–DHX36 complex have been identified as receptors for viral nucleic acids and induce type-I interferons by activating MAVS- or STING-mediated pathways *(**21**,*  *22**)*. Moreover, DHX33 functions as an RNA sensor for the NLRP3 inflammasome *(**23**)*. Upon stimulation with viral double-stranded (ds)RNA or its synthetic analogue, poly(I:C), DHX33 interacts with NLRP3 to initiate NLRP3 oligomerization and trigger the inflammasome activation. Thus, such DDX/DHX proteins are involved in the regulation of the innate immune systems. In addition to these findings, recent studies have reported that some DDX/DHX proteins (*e.g.* DDX1, DDX3X, DDX6, DDX17 and DHX36) localize to SGs in response to stress stimuli *(**24**)*, although the roles of the SG localization of these DDX/DHX proteins remain obscure. Furthermore, the list of DDX family proteins that localize to SGs is considered incomplete and requires expansion.

Here, we investigated the subcellular localization of various DDX/DHX family proteins under stress conditions and identified several DDX/DHX molecules, including DHX33 (a viral RNA sensor for the NLRP3 inflammasome), as previously unknown components of SGs. We also show that when macrophages are exposed to stress stimuli that have the potential to induce the formation of both SGs and the NLRP3 inflammasome, such as the cytoplasmic delivery of poly(I:C), macrophages preferentially assemble the NLRP3 inflammasome but not SGs. Mechanistically, we found that under such unique conditions, the NLRP3 inflammasome incorporates and sequesters the SG core protein, G3BP. Furthermore, a decrease in the intracellular K^+^ concentration, a key ‘common’ step for NLRP3 activation, also significantly inhibited SG assembly. Our results reveal the functional interplay between SG formation and NLRP3 inflammasome-mediated pyroptosis and delineate a molecular mechanism that regulates cell-fate decisions and anti-viral innate immunity under stress.

## Materials and Methods

### Cell and culture conditions

U2OS cells were gifted by Dr H. Iba (The University of Tokyo) and cultured in Dulbecco's modified Eagle's Medium (DMEM). THP-1 cells were provided by RIKEN BRC and were maintained in Roswell Park Memorial Institute (RPMI) 1640 medium. J774A.1 cells were gifted by Dr T. Ichinohe (The University of Tokyo) and were cultured in DMEM. Both DMEM and RPMI 1640 were supplemented with 10% foetal bovine serum (FBS) and l-glutamine. Cells were grown at 37°C in a humidified incubator with 5% CO_2_. To differentiate THP-1 into macrophages, THP-1 cells were stimulated with 120 nM TPA for 16 h and then cultured in fresh RPMI 1640 for 48 h *(**23**)*. For the inhibition of nigericin-mediated potassium efflux, KCl was added to culture medium free of KCl and NaCl to achieve final concentrations of 20, 40, 60 and 80 mM. For osmolarity adjustments, the combined concentration of NaCl and KCl was maintained at 114.9 mM in DMEM. U2OS cells were then cultured in this KCl-adjusted culture medium with 5 μM thapsigargin and 5 μM nigericin for 1 h.

### Plasmids and transfection

DDX5, DDX18, DDX19A, DDX24, DDX28, DHX33, DDX35, DDX41 and DDX59 were subcloned into a pcDNA4-Myc vector. Deletion mutants of DHX33 were subcloned into the pcDNA3-Flag vector to express N-terminally Flag-tagged proteins. For transient transfection, cells were cultured in 35-mm dishes and transfected with 1 μg plasmid DNA using X-tremeGENE9 DNA Transfection Reagent (Sigma-Aldrich) according to the manufacturer's instructions. Sixteen hours after transfection, cells were cultured in fresh medium for an additional 4 h before analysis.

### Immunoblotting analysis

Lysis buffer contained 20 mM Tris–HCl (pH 7.5), 1% Triton X-100, 0.5% deoxycholate (DOC), 10% glycerol, 137 mM NaCl, 2 mM EDTA, 50 mM β-glycerophosphate, 10 mM NaF, 1 mM sodium vanadate, 1 mM dithiothreitol, 1 mM phenylmethylsulfonyl fluoride, 10 μg/ml leupeptin and 10 μg/ml aprotinin. SDS-PAGE loading buffer is 65 mM Tris–HCl (pH 6.8), 5% (v/v) 2-mercaptoethanol, 3% SDS, 0.1% bromophenol blue and 10% glycerol. Cell lysates were prepared in lysis buffer, and supernatants were collected and concentrated by methanol-chloroform precipitation *(**25**)*. To 500 μl of the collected cell supernatant, 500 μl of methanol and 125 μl of chloroform were added and vortexed. This was followed by centrifugation at 15,000×g for 10 min to discard the upper liquid layer. Then, 500 μl of methanol was added, and the mixture was centrifuged again at 15,000×g for 10 min. The supernatant was removed, and the pellet was dried at 55°C. Finally, the dried pellet was dissolved in 40 μl of SDS-PAGE loading buffer and used for polyacrylamide gel electrophoresis and immunoblotting. Immunoblotting analyses were performed as previously described *(**26**)*. Images were captured using the LAS-4000 system (GE). The antibodies and their respective concentrations used were as follows: anti-FLAG M2 (Sigma-Aldrich, F1804, 1:1000), anti-NLRP3 D4D8T (Cell Signaling Technology, 15101S, 1:1000), anti-caspase-1 D7F10 (Cell Signaling Technology, 3866S, 1:1000), anti-ASC B-3 (Santa Cruz Biotechnology, sc-514414, 1:1000), anti-ASC (MBL, D086-3, 1:1000), anti-phospho-eIF2α(Ser51) D9G8 (Cell Signaling Technology, 3398S, 1:2000), anti-eIF2α D-3 (Santa Cruz Biotechnology, sc-133132, 1:2000), anti-DHX33 (Bethyl Laboratories, A300-800A, 1:10,000), and anti-β-actin (Fuji Film, 010-27841, 1:2000). We used the pre-stained protein markers, broad range (Nacalai Tesque, #02525-35) as molecular weight markers for SDS-PAGE.

### siRNA knockdown experiments

U2OS cells plated on a 6-well plate were transfected with AllStars Negative Control siRNA (QIAGEN) or siRNA targeting human DHX33 (5′-CCCAAAUGUGCUCACCUUUdTdT-3′) using Lipofectamine RNAiMAX (Invitrogen). Two days after transfection, the medium was replaced with a fresh medium, and the cells were further cultured overnight and used for the experiments.

### Immunofluorescence staining and imaging

Cells cultured on glass coverslips or glass-bottomed plates were treated with 0.3 mM sodium arsenite (SA; Fujifilm Wako, 013-15481), 5 μM thapsigargin (Fujifilm Wako, 205-17283), 43°C HS for 1 h, or were transfected with low-molecular-weight poly(I:C) (InvivoGen, tlrl-picw) to induce SG assembly. Where indicated, cells were treated with 5 μM nigericin (Sigma-Aldrich, N7143) for 1 h to induce potassium efflux. Cells were then fixed with 1% paraformaldehyde (PFA) in phosphate-buffered saline (PBS) for 10 min and permeabilized with 0.1% Triton X-100 in PBS for 10 min. After washing twice with PBS, cells were incubated in BlockAce blocking solution (Snow Brand) for 1 h at 4°C. Cells were then washed three times with PBS and incubated with primary antibodies diluted in PBS containing 2% bovine serum albumin (BSA) overnight at 4°C. The following primary antibodies were used: anti-G3BP1 23/G3BP (BD Biosciences, 611126, 1:2000), anti-G3BP2 (Abcam, ab86135, 1:5000), anti-eIF4G C45A4 (Cell Signaling Technology, #2469, 1:2000), anti-p68 RNA helicase (DDX5) D-7 (Santa Cruz Biotechnology, sc-365164, 1:200), anti-DHX33 (Bethyl Laboratories, A300-800A, 1:2000), anti-NLRP3 Cryo2 (AdipoGen Life Sciences, AG-20B-0014-C100, 1:500), anti-ASC B-3 (Santa Cruz Biotechnology, sc-514414, 1:200), anti-RPS3 2G7H4 (Proteintech, 66046-1-Ig, 1:2000), anti-RPS6 5G10 (Cell Signaling Technology, #2217, 1:500), anti-Myc 9B11 (Cell Signaling Technology, #2276, 1:8000) and anti-Flag M2 (Sigma-Aldrich, F1804, 1:1000). Cells were washed three times with PBS and then incubated with the appropriate secondary antibodies in PBS containing 2% BSA for 1 h at room temperature: Alexa Fluor 488-conjugated anti-mouse IgG (Thermo Fisher Scientific, A-11029, 1:1000), Alexa Fluor 568-conjugated anti-mouse IgG (A-11019, 1:1000), Alexa Fluor 568-conjugated anti-rabbit IgG (A-11036, 1:1000), Alexa Fluor 488-conjugated anti-rabbit IgG (Jackson ImmunoResearch, 711-545-152, 1:1000), Alexa Fluor 488-conjugated anti-mouse IgG_1_ (Thermo Fisher Scientific, A-21121, 1:1000), Alexa Fluor 568-conjugated anti-mouse IgG_2a_ (A-21134, 1:1000), Alexa Fluor 568-conjugated anti-mouse IgG_1_ (A-21124, 1:1000), Alexa Fluor 488-conjugated anti-mouse IgG_2b_ (A-21141, 1:1000), and Alexa Fluor 647-conjugated anti-Rabbit IgG (A-21246, 1:1000). For the staining of nuclei, cells were stained with 4′,6-diamidino-2-phenylindole (DAPI). Cells were washed three times with PBS and mounted in FluorSave Reagent (Merck Millipore). Images were captured using a Nikon Eclipse Ti-fluorescent microscope equipped with an ORCA-Fusion BT digital CMOS camera (Hamamatsu Photonics).

### *In situ* hybridization

Cells grown on glass coverslips were transfected with poly(I:C) (5 μg/ml) and then incubated for 2 h, followed by fixing with 1% PFA in PBS. After permeabilization with 0.1% TritonX-100 in PBS for 10 min, the cells were washed three times with 2x saline sodium citrate buffer (2xSSC) for 10 min, incubated with PerfectHyb Plus hybridization buffer (Sigma-Aldrich) for 15 min at 42°C, and further incubated with biotinylated oligo(dT) (Eurofins Genomics, custom order, 2 ng/ml) in PerfectHyb Plus buffer for 1 h at 42°C. After washing with 2×SSC, the cells were treated with 4% BlockAce for 1 h at 4°C and subsequently incubated with an antibody against G3BP1 in PBS containing 2% BSA overnight at 4 °C. Cells were then incubated with Alexa Fluor 568-conjugated anti-mouse IgG, Alexa Fluor 488-conjugated streptavidin (Thermo Fisher Scientific, S11223, 1:2000), and DAPI in PBS containing 2% BSA for 1 h. Following three PBS washes, cells on coverslips were mounted on slides using FluorSave Reagent (Merck Millipore). Images were obtained using a Nikon Eclipse Ti-fluorescent microscope equipped with ORCA-Fusion BT digital CMOS camera (Hamamatsu Photonics).

### Bioinformatic analyses

The intrinsic disorder tendency of DHX33 was analysed using IUPred3 (https://iupred3.elte.hu/) *(**27**)*. Residue with predicted scores above 0.5 is considered to be disordered. The three-dimensional structure of DHX33 was predicted by Alphafold2 *(**28**)*. The full-length amino acid sequence of human DHX33 was used as an input.

## Results

### Identification of DDX/DHX family proteins that localize to SGs

**Fig. 1:**
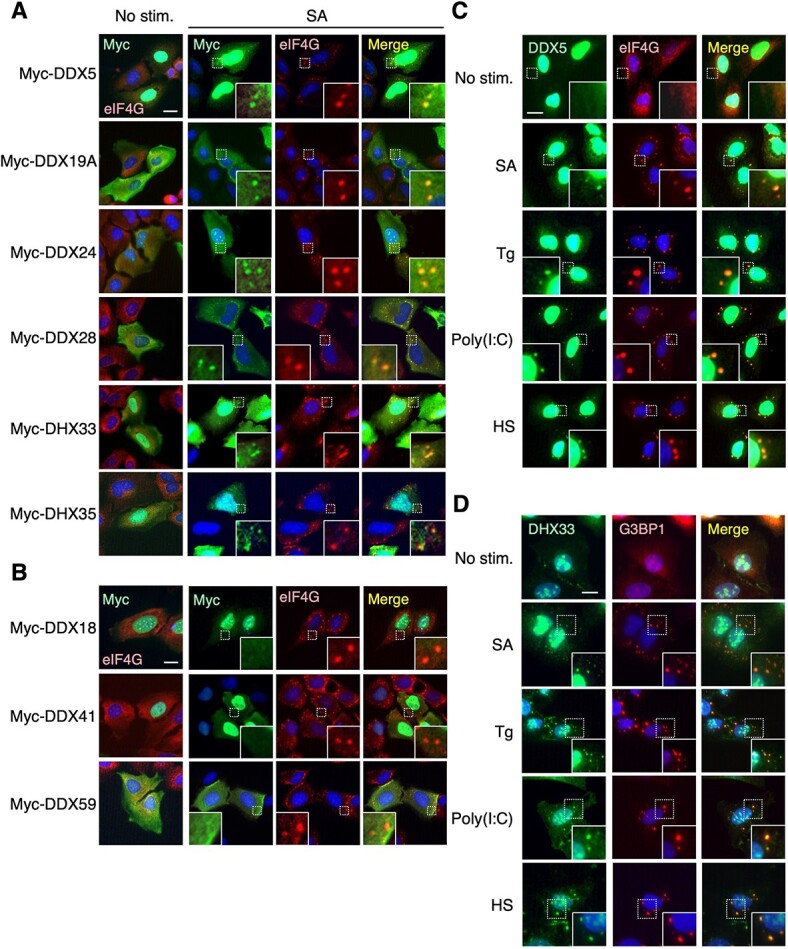
**Identification of DDX/DHX family proteins that localize to SGs**. (A, B) U2OS cells were transiently transfected with Myc-tagged DDX or DHX proteins as indicated and treated with 0.3 mM SA for 1 h. The cells were immunostained for Myc-tagged proteins (green) and the SG marker eIF4G (red). (C, D) U2OS cells were left untreated or stimulated with SA (0.3 mM for 1 h), thapsigargin (Tg, 5 μM for 1 h), poly(I:C) transfection (1.5 μg/ml for 3 h) or HS (43°C for 1 h). Endogenous DDX5 (C) or DHX33 (D) (green) and the typical SG components, eIF4G or G3BP1 (red), were detected by immunofluorescence staining using the appropriate antibodies. (A–D) Where indicated, the areas in the small squares with dashed lines were enlarged and are shown in the lower insets. Nuclei were stained with DAPI (blue). Scale bars: 20 μm.

To gain insight into the functional relevance of SG formation in the regulation of the DHX and DDX family members of RNA helicases, we initially sought to identify novel DDX/DHX family proteins that localize to SGs. Although DDX/DHX family proteins primarily reside in the nucleus, previous studies have shown that some of the family members localize not only to the nucleus but also to other cellular compartments, such as the cytoplasm and mitochondria *(**24**,*  *29**)*. Since SGs are assembled only in the cytoplasm *(**1**)*, we focused on several DDX/DHX family proteins that can reside in the cytoplasm but have not previously been associated with SGs (*i.e.* DDX5, DDX18, DDX19A, DDX24, DDX28, DHX33, DHX35, DDX41 and DDX59). To investigate whether these molecules localize to SGs, we first transiently transfected U2OS cells with a plasmid expressing one of these N-terminally Myc-tagged DDX/DHX proteins and examined their subcellular localization by immunofluorescence staining using an anti-Myc antibody. Following treatment of the transfected cells with a SG inducer, SA, Myc-tagged DDX5, DDX19A, DDX24, DDX28, DHX33 and DHX35 proteins accumulated in cytoplasmic punctate structures co-stained with a SG marker, eIF4G (Fig. 1A), whereas Myc-tagged DDX18, DDX41 or DDX59 did not (Fig. 1B). Thus, these findings suggest that the former group of DDX/DHX proteins are previously unidentified constituents of SGs.

To further determine whether the endogenous proteins are indeed recruited to SGs, we examined the subcellular localization of two representative molecules, DDX5 and DHX33, by immunofluorescence staining using antibodies specific to these proteins. Following treatment of U2OS cells with one of four inducers of SGs [*i.e.* SA, thapsigargin (Tg; an ER stressor), low-molecular-weight poly(I:C) transfection, or HS], endogenous DDX5 and DHX33 proteins accumulated into SGs under all the stress conditions tested, as assessed by co-staining with the SG markers, eIF4G or G3BP1 (Fig. 1C for DDX5 and Fig. 1D for DHX33). Thus, we concluded that these DDX/DHX family molecules are bona fide components of SGs.

### The DUF1605 domain of DHX33 is crucial for its SG localization.

**Fig. 2:**
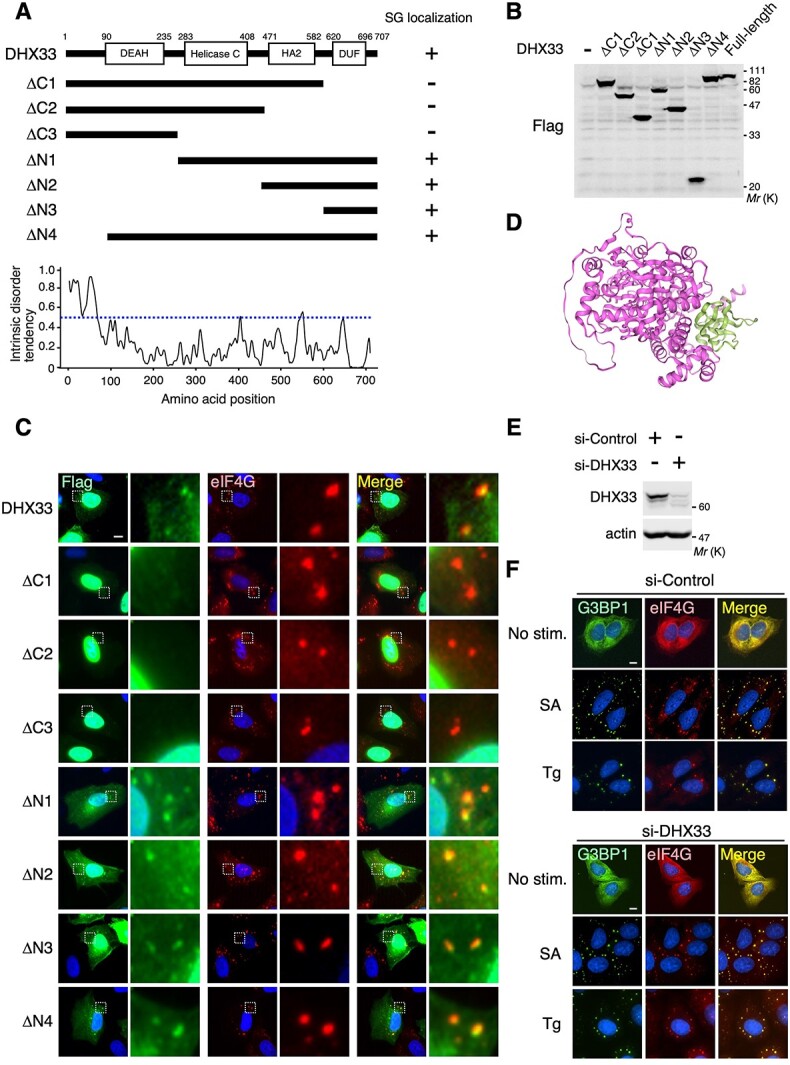
**The DUF1605 domain is crucial for SG localization of DHX33.** (A) The domain structure of DHX33 and the DHX33 deletion constructs used (upper). The intrinsically disordered tendency of DHX33 was predicted using an IUPred3 algorithm (lower graph). Residues with predicted scores above 0.5 (blue dotted line) were considered to be disordered. (B) U2OS cells were transiently transfected with Flag-tagged DHX33 (full-length or its deletion mutants) as indicated. The expression levels of the indicated proteins were analysed by immunoblotting with an anti-Flag antibody. (C) U2OS cells were transiently transfected with Flag-DHX33 (full-length or its deletion mutants), and treated with SA (0.3 mM for 1 h). The localization of Flag-DHX33 (green) and endogenous eIF4G (red) was assessed by immunofluorescence staining. The areas in the small squares in the left panels were enlarged and are shown in the right panels. Blue, DAPI. Scale bar, 10 μm. (D) The three-dimensional structure of DHX33 predicted by Alphafold2. The DUF1605 domain is shown in green. (E, F) U2OS cells were transfected with siRNA targeting DHX33 (si-DHX33) or with control siRNA (si-Control). (E) Depletion of DHX33 protein was monitored by immunoblotting using an anti-DHX33 antibody. Actin served as a loading control. (F) U2OS cells transfected with either si-Control (upper) or si-DHX33 (lower) were treated with SA (0.3 mM for 1 h) or Tg (5 μM for 1 h), and immunostained for SG marker proteins, G3BP1 (green) and eIF4G (red). Blue, DAPI. Scale bars: 10 μm.

Recent studies have shown that, besides its role in RNA metabolism, DHX33 functions as a cytosolic viral RNA sensor and promotes an immune response *(**23**,*  *30**)*. While SGs regulate anti-viral innate immunity by incorporating various cytoplasmic immune-related molecules *(**7**,*  *31**)*, the functional interplay, if any, between SG formation and DHX33 remains unknown. Therefore, of the newly identified SG component proteins, we further analysed DHX33. DHX33 is composed of several structural domains: DEAH-box (DEAH), helicase C-terminal domain (Helicase-C), helicase-associated domain 2 (HA2), and domain of unknown function 1605 (DUF1605) (Fig. 2A) *(**32**)*. To identify the site in DHX33 essential for its SG localization, we constructed a series of deletion mutants of DHX33 and evaluated their localization in SGs following SA treatment. Initially, we confirmed by western blot analysis that all the Flag-tagged deletion mutants were expressed at a level comparable to full-length Flag-DHX33 (Fig. 2B). Immunofluorescence microscopy analysis revealed that the DHX33 constructs lacking the carboxy-terminal DUF1605 domain (*i.e.* ΔC1-3) failed to localize in SGs (Fig. 2A and C). Conversely, the ΔN3 mutant containing only the DUF1605 domain was able to localize in SGs. Thus, the DUF1605 domain, but not the other domains in DHX33, was necessary and sufficient for DHX33 localization in SGs. Since many SG component proteins localize in SGs through IDR-mediated protein–protein interactions (PPIs) *(**6**)*, we next analysed the degree and distribution of IDRs present in DHX33 using an IUPred3 algorithm *(**27**)*. The DUF1605 domain of DHX33, however, did not possess IDRs of an appropriate length (Fig. 2A, lower graph). The prediction of a three-dimensional structure by Alphafold2 indicated that the DUF1605 domain was predominantly exposed on the surface of the DHX33 protein (Fig. 2D). Therefore, although the precise function of the DUF1605 domain remains to be elucidated, DHX33 is likely recruited to SGs independently of IDRs by interacting with other SG component proteins via its DUF1605 domain. Indeed, we observed that the depletion of DHX33 by its specific siRNA did not affect SA- or Tg-induced SG assembly in U2OS cells (Fig. 2E and F), suggesting that DHX33 is passively recruited to SGs.

### Cytoplasmic delivery of poly(I:C) preferentially induces the formation of the NLRP3 inflammasome but not of SGs in macrophages

**Fig. 3:**
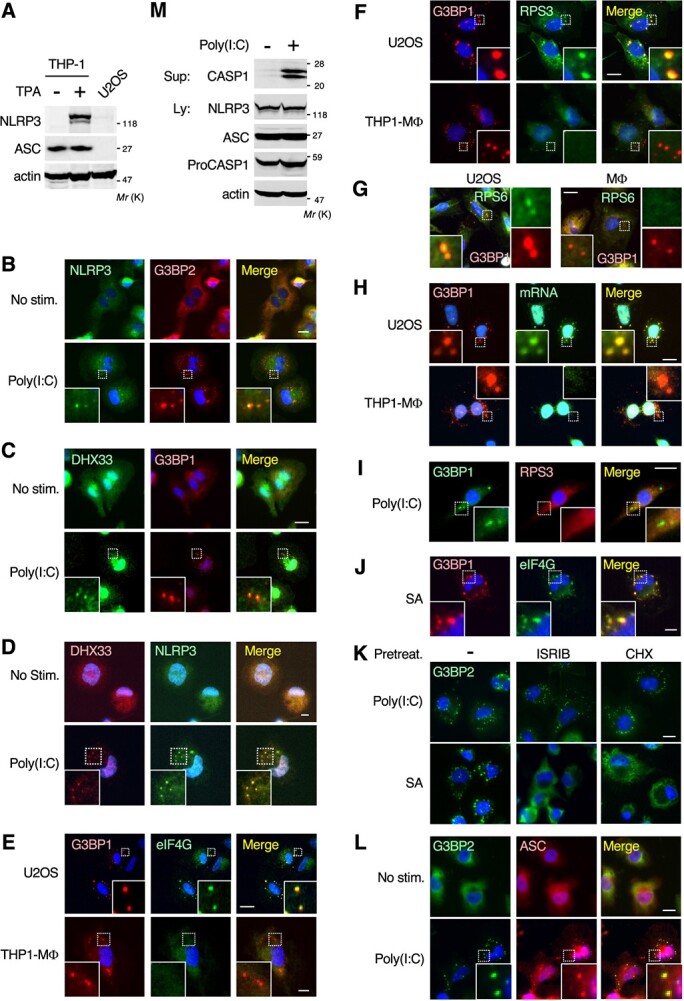
**Cytoplasmic delivery of poly(I:C) preferentially induces the formation of the NLRP3 inflammasome but not of SGs in macrophages**. (A) Human THP-1 monocytes were differentiated into macrophages by TPA treatment. The expression levels of NLRP3 and ASC in parental THP-1 cells, THP-1-derived macrophages, and U2OS cells were assessed by immunoblotting using the appropriate antibodies. Actin served as a loading control. (B–D) THP-1-derived macrophages were transfected with poly(I:C) (5 μg/ml for 2 h), and immunostained for endogenous NLRP3, DHX33, and G3BP1/2 as indicated. (B, C) Green, NLRP3 or DHX33; red, G3BP1 or G3BP2. (D) Green, NLRP3; red, DHX33. (E–G) U2OS cells or THP-1-derived macrophages (THP1-MΦ) were transfected with poly(I:C) and stained for SG component proteins, G3BP1, eIF4G and RPS3, or RPS6. (E) Red, G3BP1; green, eIF4G. (F, G) Red, G3BP1; green, RPS3 or RPS6. (H) The indicated cells were transfected with poly(I:C), and then mRNA (green) was visualized by *in situ* hybridization using an oligo-dT probe. The cells were also stained for G3BP1 (red). (I) Murine J774A.1 macrophages were transfected with poly(I:C) (5 μg/ml for 2 h) and stained for endogenous G3BP1 (green) and RPS3 (red). (J) THP1-derived macrophages were treated with SA (0.3 mM for 1 h) and stained for G3BP1 (red) and eIF4G (green). (K) Macrophages were pretreated with 2 μg/ml ISRIB or 15 μg/ml cycloheximide (CHX) for 2 h before treatment with poly(I:C) transfection (5 μg/ml for 2 h) or SA (0.3 mM for 1 h). The cells were stained for G3BP2 (green). (L) Macrophages were transfected with poly(I:C) and stained for G3BP2 (green) and ASC (red). (B–L) Blue, DAPI. Scale bars, 10 μm. (M) Macrophages were transfected with poly(I:C) (5 μg/ml for 6 h) and then cell culture supernatants (Sup) and cell lysates (Ly) were collected. Cell culture supernatants were concentrated by methanol-chloroform precipitation and subjected to immunoblotting for caspase-1 (CASP1). Cell lysates were also analysed by immunoblotting using the appropriate antibodies.

In prior sections, we demonstrated that DHX33 localized to SGs under various stress conditions, including the cytosolic delivery of poly(I:C). However, DHX33 has also been shown to serve as a component of the NLRP3 inflammasome in macrophages, and plays an essential role in its assembly and activation induced by poly(I:C) *(**23**)*. Therefore, in theory, poly(I:C) transfection would simultaneously induce the formation of both SGs and the NLRP3 inflammasome in macrophages, and thus these two organelles may compete for DHX33. In this regard, however, a recent study showed that the preformation of SGs by SA treatment inhibited nigericin-induced assembly of the NLRP3 inflammasome in macrophages *(**13**)*. Nevertheless, it remains unknown whether poly(I:C) transfection, which serves as an inducer of both organelles, leads to the formation of SGs or the NLRP3 inflammasome (or both) in macrophages. To address this question, we next investigated the effect of poly(I:C) on the assembly of these two organelles in macrophages. For this purpose, we differentiated the human monocyte cell line THP-1 into macrophages by TPA treatment and used these macrophages as a model *(**23**)*. Initially, we confirmed that, consistent with the fact that NLRP3 is mainly expressed in differentiated macrophages, its expression was detected only when THP-1 cells were primed with TPA (Fig. 3A). These THP-1-derived macrophages were then transfected with poly(I:C) and immunostained for NLRP3 and G3BP2 to monitor the formation of the NLRP3 inflammasome and SGs, respectively (Fig. 3B). Following poly(I:C) transfection, NLRP3 formed cytoplasmic punctate foci, indicating the assembly and activation of the NLRP3 inflammasome. Furthermore, poly(I:C) also triggered the accumulation of the SG marker protein, G3BP, into cytoplasmic granular foci, implying the formation of SGs. Interestingly, however, we found that these G3BP-positive foci merged with the NLRP3-positive foci. Thus, NLRP3 and G3BP colocalized in cytoplasmic punctate structures in poly(I:C)-stimulated macrophages. Consistent with their colocalization, DHX33 was co-stained not only with NLRP3 but also with G3BP at these cytoplasmic foci following poly(I:C) transfection (Fig. 3C and D). Therefore, these findings raise at least two possibilities: (i) Poly(I:C) transfection simultaneously induces the formation of both the NLRP3 inflammasome and SGs, and these two organelles merge together in macrophages; or (ii) poly(I:C) transfection preferentially induces the assembly of the NLRP3 inflammasome rather than SGs, and the inflammasome selectively incorporates and sequesters the SG component protein, G3BP. To distinguish between these two possibilities, we further analysed components of the poly(I:C)-induced, NLRP3/G3BP double-positive foci in THP-1-derived macrophages. Since SGs consist of multiple components other than G3BP, including translation initiation factors (*e.g.* eIF4G), 40S ribosomal proteins (*e.g.* RPS3 and RPS6), and various mRNAs *(**1**)*, we tested if the observed foci contained these key SG marker proteins and mRNAs by immunofluorescence staining and by *in situ* hybridization using an oligo-dT probe, respectively. Initially, as a control experiment, we transfected poly(I:C) into U2OS cells, which express neither NLRP3 nor the inflammasome adaptor protein ASC (see Fig. 3A), and are thus defective in NLRP3 inflammasome assembly. We showed that all the key SG constituents, *i.e.* eIF4G (Fig. 3E, top), RPS3 (Fig. 3F, top), RPS6 (Fig. 3G, left), and mRNAs (Fig. 3H, top), accumulated in G3BP-positive foci. Therefore, in line with previous reports *(**3**,*  *33**)*, poly(I:C) transfection elicited the formation of canonical SGs in NLRP3-deficient U2OS cells. However, in macrophages, the G3BP-positive foci induced by poly(I:C) were devoid of all the SG components tested. No eIF4G, RPS3, RPS6 or mRNAs were detected in the foci (Fig. 3E and F, bottom; Fig. 3G, right; Fig. 3H, bottom). Similar results were also obtained with murine J774A.1 macrophages: After poly(I:C) transfection, the SG marker protein RPS3 did not localize to G3BP-positive foci (Fig. 3I), indicating that this phenomenon is conserved across species. In contrast to poly(I:C) transfection, we showed that SA treatment (a SG inducer) induced the assembly of canonical SGs in THP-1-derived macrophages, as the key SG component, eIF4G, accumulated in G3BP-positive foci (Fig. 3J). Thus, these findings indicate that the poly(I:C)-induced, G3BP-positive foci observed in macrophages are not SGs. Consistently, treatment of macrophages with one of two chemical inhibitors of SG formation, integrated stress response inhibitor (ISRIB) or cycloheximide (CHX) *(**1**,*  *34**)*, did not suppress the poly(I:C)-induced assembly of these G3BP-positive foci (Fig. 3K), further supporting this conclusion.

**Fig. 4:**
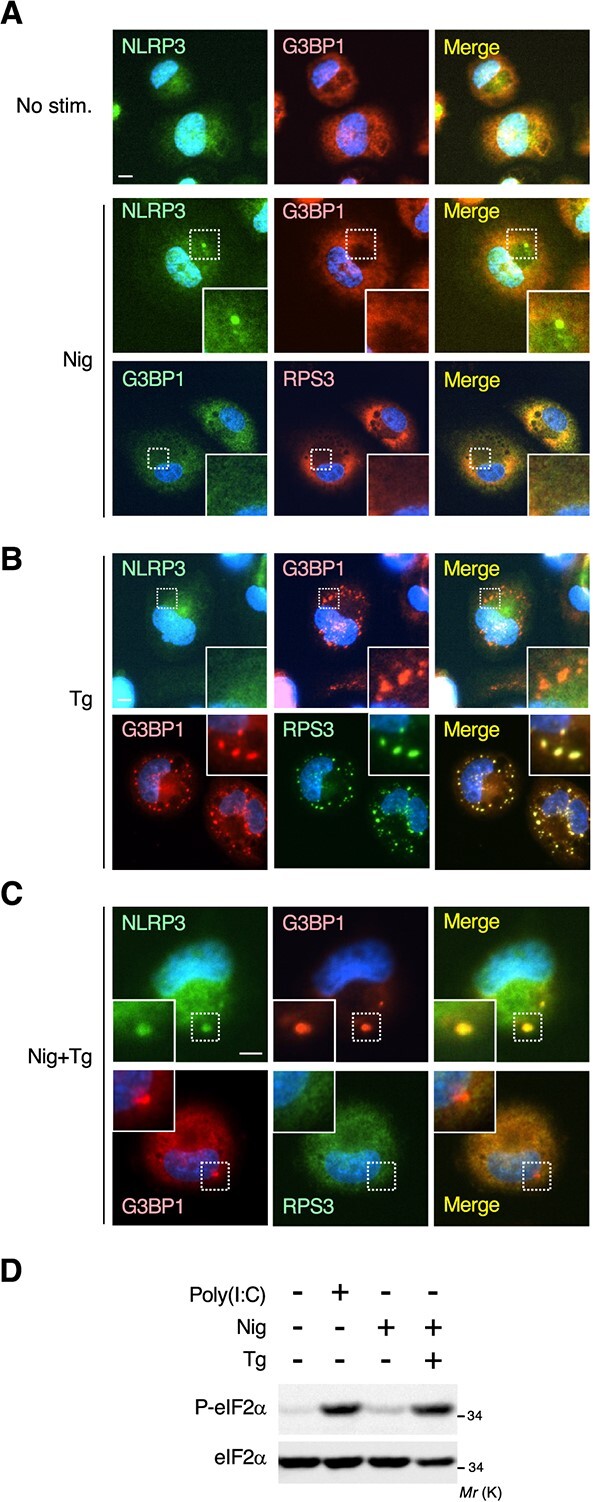
**The NLRP3 inflammasome incorporates G3BP in a stimulus-dependent manner**. (A–C) Macrophages were treated with nigericin (Nig; 5 μM for 1 h) (A) or thapsigargin (Tg; 5 μM for 1 h) (B) alone, or with both simultaneously (C). Cells were then fixed and stained for NLRP3, G3BP1 and RPS3. (A) Green, NLRP3 or G3BP1; red, G3BP1 or RPS3. (B, C) Green, NLRP3 or RPS3; red, G3BP1. Blue, DAPI. Scale bars, 10 μm. (D) THP-1-derived macrophages were transfected with poly(I:C) (5 μg/ml for 2 h), or were treated with Nig (5 μM for 1 h) alone, or Nig together with Tg (5 μM for 1 h) as indicated. Cell extracts were probed for phosphorylated eIF2α (P-eIF2α; upper). The same filter was then stripped and reprobed for total eIF2α (lower).

Next, to test whether the observed foci in macrophages are the NLRP3 inflammasome, we examined if they contained the inflammasome marker protein, ASC. As shown in Fig. 3L, after poly(I:C) transfection, ASC accumulated in the poly(I:C)-induced foci. Moreover, poly(I:C) stimulation resulted in the release of cleaved caspase-1 into the cell culture supernatant (Fig. 3M), which is a hallmark of NLRP3 inflammasome activation *(**23**)*. Based on these combined findings, we concluded that cytoplasmic delivery of poly(I:C) preferentially triggers the assembly of the NLRP3 inflammasome, but not SGs, in macrophages. Thus, DHX33 is primarily employed as a cytosolic dsRNA sensor for the inflammasome. Furthermore, we also found that, under these conditions, the SG core proteins, G3BP1/2, were incorporated into the NLRP3 inflammasome. Given that G3BP1/2 are essential for the assembly of SGs induced by various stresses, including poly(I:C) transfection *(**3**,*  *35**)*, the efficient sequestration of G3BP1/2 proteins into the NLRP3 inflammasome at least partially explains why poly(I:C) transfection fails to induce SG assembly in macrophages.

### The NLRP3 inflammasome incorporates G3BP in a stimulus-dependent manner

Since we observed that the poly(I:C)-induced NLRP3 inflammasome sequestered the SG core protein G3BP, we next tested whether the NLRP3 inflammasome induced by stimuli other than poly(I:C) could also incorporate G3BP. For this purpose, THP-1-derived macrophages were stimulated with the well-known inflammasome inducer, nigericin (Nig), and the subcellular localization of G3BP was then monitored by fluorescence microscopy. As anticipated, nigericin treatment led to the formation of the NLRP3 inflammasome, as assessed by the accumulation of NLRP3 into a cytoplasmic punctate structure (Fig. 4A, middle). However, interestingly, under these conditions, G3BP did not colocalize with the NLRP3 inflammasome, but instead remained diffusely distributed in the cytoplasm, as did another SG component protein, RPS3 (Fig. 4A, middle and bottom). Thus, unlike the poly(I:C)-induced NLRP3 inflammasome, nigericin-induced one did not incorporate G3BP. These findings indicate that the inflammasome-mediated G3BP sequestration occurs in a stimulus-dependent manner.

What then is the difference between poly(I:C) and nigericin stimulation in controlling the inflammasome recruitment of G3BP? Considering that poly(I:C) stimulation has the potential to induce both NLRP3 inflammasome and SG formation, we hypothesized that the simultaneous application of inflammasome- and SG-inducing stimuli might lead to the incorporation of G3BP into the inflammasome in macrophages. To test this, we explored whether the concurrent treatment of macrophages with nigericin and Tg (an inducer of ER stress and SG formation) would lead to the localization of G3BP within the NLRP3 inflammasome. Initially, we observed that the treatment of macrophages with Tg alone did not induce the NLRP3 inflammasome (Fig. 4B, upper) but did elicit the formation of canonical SGs, as assessed by the colocalization of the SG marker protein, RPS3, into G3BP1-positive foci (Fig. 4B, lower). THP-1-derived macrophages were then simultaneously stimulated with nigericin and Tg, and the subcellular localization of NLRP3 and G3BP was monitored by immunofluorescence staining. Intriguingly, we found that, similar to poly(I:C) stimulation, combined treatment with both agents promoted the assembly of the NLRP3 inflammasome (Fig. 4C, upper), with G3BP (but not RPS3) being incorporated into the inflammasome (Fig. 4C, lower). We showed that the concurrent treatment with nigericin and Tg induced eIF2α phosphorylation, which is necessary for SG formation, as efficiently as poly(I:C) transfection (Fig. 4D). These results indicate that when macrophages are exposed to stimuli capable of inducing the formation of both the NLRP3 inflammasome and SGs (*e.g.* cytoplasmic dsRNA, and a combination of nigericin with Tg), macrophages preferentially form the NLRP3 inflammasome and avoid SG formation, at least in part, by sequestering the SG core protein, G3BP, into the inflammasome.

### Nigericin-mediated potassium efflux inhibits SG assembly independently of NLRP3 inflammasome formation

**Fig. 5:**
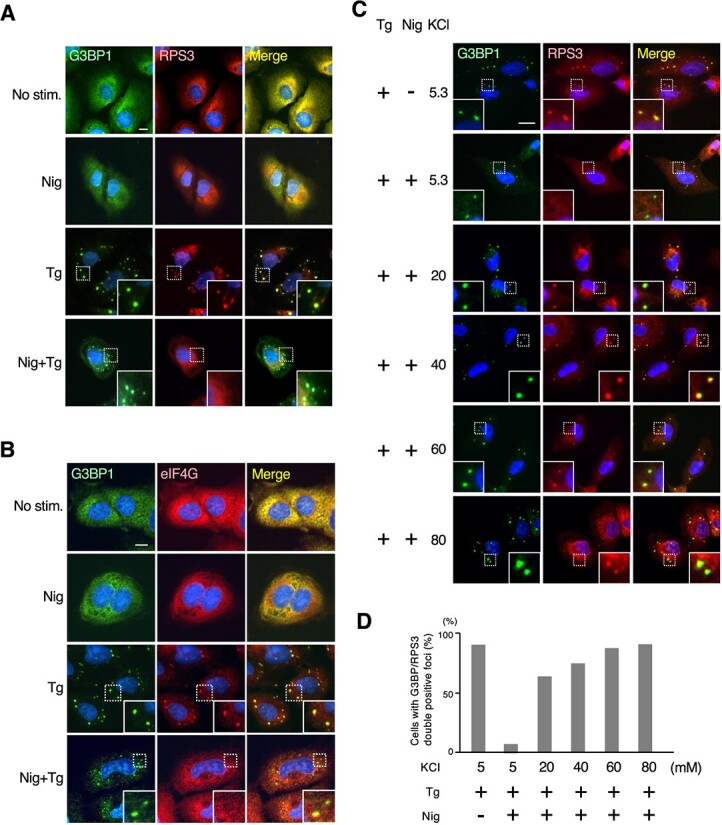
**Nigericin-mediated potassium efflux inhibits SG assembly independently of NLRP3 inflammasome formation.** (A, B) U2OS cells were treated with nigericin (5 μM for 1 h) or thapsigargin (5 μM for 1 h) alone, or with both simultaneously as indicated. Cells were stained for G3BP1 (green) and the other SG component proteins, RPS3 (A) and eIF4G (B) (red). (C, D) U2OS cells were treated with thapsigargin (5 μM), together with or without nigericin (5 μM) for 1 h in culture medium containing different concentrations of KCl (5.3, 20, 40, 60 or 80 mM) as indicated. Cells were immunostained for G3BP1 (green) and RPS3 (red) (A), and the percentage of cells with G3BP/RPS3 double-positive foci per cells with G3BP-positive foci was determined and shown as a bar graph. More than 100 cells were counted per sample. (A–C) Blue, DAPI. Scale bars: 20 μm.

In relation to the above analysis, we also found that nigericin treatment prevented SG formation not only by inducing NLRP3 inflammasome formation but also by an inflammasome-independent mechanism. In inflammasome-deficient U2OS cells, Tg treatment efficiently induced the assembly of SGs where G3BP colocalized with RPS3 (Fig. 5A) as well as eIF4G (Fig. 5B). However, when U2OS cells were co-stimulated with Tg and nigericin, although G3BP1 still accumulated in cytoplasmic punctate structures, the key SG component proteins (*i.e.* RPS3 and eIF4G) remained diffusely distributed in the cytoplasm and were thus excluded from the G3BP-positive spots. Therefore, in the presence of nigericin, Tg treatment failed to induce the assembly of canonical SGs, even though U2OS cells are defective in NLRP3 inflammasome formation, suggesting the existence of another, as yet unidentified, mechanism for nigericin-mediated inhibition of SG assembly. Previous studies have demonstrated that SG formation is initiated by oligomerization of the SG core protein G3BP *(**2**,*  *4**)*. Therefore, our results suggest that nigericin treatment has a relatively minor effect on the Tg-induced initial assembly of G3BP oligomers but efficiently inhibits subsequent processes of SG formation, such as the LLPS-mediated recruitment of various RNPs.

We then investigated how nigericin inhibited the assembly of canonical SGs independently of NLRP3 inflammasome formation in U2OS cells. Because nigericin induces K^+^ efflux across the plasma membrane by acting as a K^+^ ionophore and leads to a substantial reduction in intracellular K^+^ levels *(**14**,*  *36**)*, and because the salt concentration is a crucial factor in determining the efficiency of LLPS droplet formation *(**37**,*  *38**)*, we hypothesized that the nigericin-induced reduction of intracellular K^+^ levels might impede the LLPS-mediated assembly of SGs. In this regard, previous studies have demonstrated that nigericin-induced K^+^ efflux can be counteracted by increasing the extracellular K^+^ concentration *(**14**,*  *17**)*. Therefore, to test this possibility, we cultured U2OS cells in standard medium (KCl; 5.3 mM) or in medium with increasing concentrations of K^+^ (KCl; 20, 40, 60 and 80 mM), wherein NaCl was iso-osmotically replaced with KCl (see Materials and Methods), and then stimulated the cells with Tg and/or nigericin (Fig. 5C and D). Under conditions of a standard extracellular K^+^ concentration (5.3 mM), co-treatment of U2OS cells with Tg and nigericin did not lead to SG formation, as assessed by the lack of RPS3 incorporation into G3BP1-positive foci. However, notably, under conditions of high extracellular K^+^ concentrations (which prevent nigericin-induced K^+^ efflux), RPS3 efficiently colocalized in G3BP1-positive foci in a manner roughly dependent on the extracellular K^+^ concentration (Fig. 5D). Thus, when K^+^ efflux was inhibited, Tg readily induced the formation of canonical SGs, even in the presence of nigericin, indicating that a reduction in intracellular K^+^ concentration suppresses SG assembly. Collectively, these findings indicate that nigericin inhibits SG formation not only by NLRP3 inflammasome formation in macrophages but also through an inflammasome-independent, more universal mechanism in various types of cells: the reduction of intracellular K^+^ levels by nigericin inhibits SG assembly most likely by impeding LLPS-mediated recruitment of key SG components, such as RPS3 and eIF4G, to G3BP-containing core condensates. Since K^+^ efflux is a common step that is essential for NLRP3 inflammasome activation induced by various stimuli *(**14**,*  *17**,*  *36**,*  *39**)*, this mechanism also likely contributes to the NLRP3 inflammasome-mediated suppression of SG assembly in macrophages.

## Discussion

In this study, we analysed the subcellular localization of several DDX/DHX family proteins under stress conditions and found that specific members of this family (*i.e.* DDX5, DDX19A, DDX24, DDX28, DHX33 and DHX35, but not DDX18, DDX41 or DDX59) were previously unidentified components of SGs. Previous studies have shown that the DDX/DHX family of RNA helicases is involved in various aspects of RNA metabolism *(**40*–*43**)*. In addition, several members of this family, including DDX5, DDX19A and DHX33, play important roles in anti-viral innate immune responses by acting as sensors for viral nucleic acids *(**20**,*  *30**,*  *44**,*  *45**)*. Therefore, the incorporation of DDX/DHX proteins into SGs may at least partially modulate various biological processes in which these molecules are involved. Indeed, SG formation has been shown to regulate innate immune responses by recruiting various signalling molecules, including RIG-I-like receptors (*e.g.* RIG-I/DDX58 and MDA-5) and others *(**7**,*  *12**,*  *31**)*. Our data suggest that SGs incorporate a greater variety of signalling molecules that regulate immune responses and cellular functions than previously thought.

Of these DDX/DHX proteins newly identified as SG components, we focused on DHX33 because of its unique function as a cytosolic viral dsRNA sensor for NLRP3 inflammasome activation *(**23**)*. Previous studies have reported that many (if not all) SG component proteins localize in SGs through IDR-mediated PPIs *(**6**)*. We revealed, however, that the DUF1605 domain of DHX33 was responsible for its SG localization, even though this domain does not possess IDRs. Therefore, although further studies are needed, DHX33 is likely recruited to SGs independently of IDRs by interacting with other SG component proteins via its DUF1605 domain. Indeed, SGs can incorporate several molecules that bind to DHX33 (*e.g.* CALML5, CRYAB, ELAVL1, HNRNPH1, PKP1, PTGES3, SQSTM1 and TRIM25) *(**2**,*  *46**,*  *47**)*. Since the DUF1605 domain is highly conserved in DHX33 orthologues in vertebrates, SG-mediated sequestration of DHX33 might be similarly conserved across species.

More importantly, we demonstrated here that, when macrophages are exposed to stress stimuli with the potential to induce the formation of both the NLRP3 inflammasome and SGs [*e.g.* poly(I:C) transfection and a combination of nigericin and Tg], macrophages preferentially assemble the NLRP3 inflammasome but avoid SG formation, and therefore, DHX33 is primarily utilized as a cytosolic dsRNA sensor for the inflammasome. Notably, we also showed that, under such unique conditions, the SG core protein, G3BP, was efficiently incorporated into the inflammasome. Since G3BP is essential for SG assembly under various stress conditions, including poly(I:C) transfection and Tg treatment *(**3**,*  *35**)*, sequestration of G3BP into the inflammasome at least partially explains why macrophages prioritize the formation of the NLRP3 inflammasome but not of SGs. In contrast, when macrophages were stimulated with nigericin alone (a stimulus that induces the formation of the NLRP3 inflammasome but not SGs), the assembled inflammasome did not incorporate G3BP, indicating that the accumulation of G3BP into the inflammasome occurs in a stimulus-dependent manner. Thus, our results suggest that the inflammasome-mediated sequestration of G3BP serves as a biological strategy that preferentially promotes the inflammasome-mediated innate immune response, particularly when macrophages are confronted with specific stimuli capable of inducing both the NLRP3 inflammasome and SGs (*e.g.* viral infection). In this context, a recent study reported that the NLRP3 inflammasome induced by nigericin incorporates DDX3X, a molecule known to contribute to SG assembly, into the inflammasome *(**13**)*. Therefore, together with our present findings, the NLRP3 inflammasome sequesters not only DDX3X but also the potent SG-nucleating protein, G3BP, to reliably inhibit the assembly of pro-survival SGs and to further promote inflammasome formation and consequent pyroptotic cell death under specific stress conditions.

Another interesting finding of this study is that a reduction in intracellular K^+^ levels inhibits SG assembly independently of NLRP3 inflammasome formation. Several lines of evidence demonstrate that salt concentration critically influences the efficiency of LLPS droplet formation, at least *in vitro (**37**,*  *38**,*  *48**,*  *49**)*. Since K^+^ is the primary intracellular cationic salt, its reduction by nigericin likely attenuates LLPS of biomolecules in cells, thereby impeding SG formation. Consistent with this notion, previous studies showed that various Na^+^/K^+^-ATPase pump inhibitors (*e.g.* digitoxin, ouabain and proscillaridin A), which decrease intracellular K^+^ levels, exert an inhibitory effect on SG formation in various cell types *(**50**,*  *51**)*. Although the precise mechanisms of action for these drugs in the regulation of SG assembly remain unknown, our results suggest that perturbation of intracellular K^+^ levels by these drugs inhibits LLPS-mediated SG assembly. In this context, in macrophages, many stimuli that induce NLRP3 inflammasome formation, such as nigericin, ATP and crystal molecules, are known to trigger K^+^ efflux *(**14**,*  *17**,*  *36**,*  *39**)*. Moreover, NLRP3 inflammasome formation itself further reduces the intracellular K^+^ concentration via gasdermin D- or pannexin 1-mediated pore formation on the plasma membrane *(**18**,*  *52**,*  *53**)*. Thus, these findings suggest that the formation of the NLRP3 inflammasome inhibits SG assembly not only by sequestering SG-nucleating proteins (G3BP1/2 and DDX3X) but also by decreasing intracellular K^+^ levels. NLRP3 inflammasome-mediated inhibition of pro-survival SG formation through these multiple mechanisms will dictate cell-fate decisions under stress conditions, including viral infection, and ensure that macrophages undergo pyroptotic cell death to promote pro-inflammatory cytokine production and the resulting anti-viral immune response.
